# Glutamate Neurotoxicity and Destruction of the Blood–Brain Barrier: Key Pathways for the Development of Neuropsychiatric Consequences of TBI and Their Potential Treatment Strategies

**DOI:** 10.3390/ijms23179628

**Published:** 2022-08-25

**Authors:** Benjamin F. Gruenbaum, Alexander Zlotnik, Ilya Fleidervish, Amit Frenkel, Matthew Boyko

**Affiliations:** 1 Department of Anesthesiology and Perioperative Medicine, Mayo Clinic, Jacksonville, FL 32224, USA; 2 Department of Anesthesiology and Critical Care, Soroka University Medical Center, Ben-Gurion of the Negev, Beer-Sheva 84105, Israel; 3 Department of Physiology and Cell Biology, Faculty of Health Sciences and Zlotowski Center for Neuroscience, Ben-Gurion University of the Negev, Beer-Sheva 84105, Israel

**Keywords:** blood–brain barrier (BBB), blood glutamate scavengers, glutamate, neuropsychiatric conditions, traumatic brain injury (TBI)

## Abstract

Traumatic brain injury (TBI) is associated with significant cognitive and psychiatric conditions. Neuropsychiatric symptoms can persist for years following brain injury, causing major disruptions in patients’ lives. In this review, we examine the role of glutamate as an aftereffect of TBI that contributes to the development of neuropsychiatric conditions. We hypothesize that TBI causes long-term blood–brain barrier (BBB) dysfunction lasting many years and even decades. We propose that dysfunction in the BBB is the central factor that modulates increased glutamate after TBI and ultimately leads to neurodegenerative processes and subsequent manifestation of neuropsychiatric conditions. Here, we have identified factors that determine the upper and lower levels of glutamate concentration in the brain after TBI. Furthermore, we consider treatments of disruptions to BBB integrity, including repairing the BBB and controlling excess glutamate, as potential therapeutic modalities for the treatment of acute and chronic neuropsychiatric conditions and symptoms. By specifically focusing on the BBB, we hypothesize that restoring BBB integrity will alleviate neurotoxicity and related neurological sequelae.

## 1. Introduction

Traumatic brain injury (TBI) has long-term cognitive and psychiatric effects, including depression, anxiety, and aggression. While the condition’s psychiatric symptoms may at first be attributable to the emotional burdens of TBI-related physical disability, it has been established that neuropsychiatric symptoms, such as memory and cognitive impairment, anxiety, depression, social withdrawal, or aggression [1,2,3], can persist for decades [4,5] after the initial brain injury. These symptoms can delay rehabilitation and a return to employment [6]. The associated psychiatric symptoms are not correlated with severity of the initial injury or with pain [7,8,9].

An association between TBI and the development of a wide range of neuropsychiatric diseases has been well established [1,2,3,10,11]. A possible explanation for the increase in the incidence of neuropsychiatric illness after TBI lies in the general mechanisms underlying the development of these diseases. However, to date, there is no unified theory that explains this phenomenon. In this review, we hypothesize that long-term dysregulation of glutamate as a result of TBI, modulated by variability in blood–brain barrier (BBB) permeability, causes a process of neurodegeneration, which ultimately leads to the development of neuropsychiatric consequences of TBI. Here, we detail the proposed pathways of the process and provide evidence for this hypothesized relationship.

## 2. Pharmacological Basis of Dementia, Anxiety, and Mood Disorders: The Role of Glutamate

The relationship between depression, anxiety, dementia, aggression, and glutamate [12,13,14] is well studied and documented in the scientific literature [3,15,16]. Traditionally, anxiety and mood disorders are treated with medications that act primarily on monoamines [17], benzodiazepines [18,19], or cannabidiol [20]; however, there is a burgeoning interest in the role of the major excitatory neurotransmitter glutamate in the etiology and treatment of anxiety [13,21,22,23], mood disorders, and disorders of social behavior and aggression [24]. Medications that attenuate glutamate release or block N-methyl-D-aspartate (NMDA) receptors [21,24,25,26] are used to treat anxiety disorders. Glutamate is the major excitatory neurotransmitter in the brain, essential for long-term potentiation and mediating long-term depression, and provides the molecular and cellular mechanisms of learning [27,28,29]. Poor long-term cognitive evaluation in patients with TBI was correlated with higher levels of brain glutamate recorded one week [30] and one month after the injury [31], confirming the link between glutamate excess and deficits in higher cognitive function. Glutamate excess following loss of neurons and astrocytes is common in stroke, dementia, and neurogenerative diseases, and after injury such as TBI [32,33].

## 3. TBI and Glutamate Dysregulation

Glutamate is the most abundant free amino acid in the brain [34]. Glutamate concentrations in the plasma and whole blood are 50–100 μM/L and 150–300 μM/L, respectively [12]; in the whole brain, they are 10,000–12,000 μM/kg [35], but they are only 1–10 μM/L in extracellular fluids (ECF) [12]. The gradient between brain cells, blood, and ECF is maintained by facilitative and active transport systems of the BBB [35]. A healthy BBB effectively prevents glutamate from moving between the intraparenchymal and blood compartments [36]. There are a number of mechanisms that cause an increase in brain glutamate associated with TBI: (i) neuronal death [37]; (ii) inflammation [38,39,40,41,42]; (iii) impaired glutamatergic recycling and signaling [43]; (iv) prolonged stress [29]; (v) astrocytic release of adenosine triphosphate (ATP) [44]; and (vi) other sources of elevated intraparenchymal glutamate [36,45]. Above all, however, we find the mechanisms of BBB destruction to be a significant factor in increased brain glutamate and its association with TBI [35].

### 3.1. The Mechanisms of Increase in Brain Glutamate Is Associated with Neuronal Death

The well-known process of neuronal death, which begins in the first minutes after a traumatic brain injury, can potentially lead to an increase in the glutamate levels in cerebrospinal fluid (CSF) and extracellular fluid (ECF) of the brain to the levels of glutamate in the whole brain, up to 10,000–12,000 µM/kg [12]. However, in practice, such high levels of glutamate in CSF and ECF of the brain do not occur, and glutamate concentrations are usually limited to about 20–50 µM/L [46,47,48,49,50]. The explanation for this phenomenon is that even with the complete destruction of the BBB, concentrations of glutamate in the CSF and ECF of the brain cannot exceed the concentration of plasma glutamate, which is approximately 50–100 µM/L [12]. Plasma effectively clears out excess glutamate, sending it into other organs and even red blood cells. Due to this process, a concentration of glutamate at 100 µM/L, at the upper level of plasma concentration, is the maximum that can be recorded in the CSF and ECF of the brain. Rare exceptions may occur when whole blood is included in CSF collection.

However, the concentration of glutamate can reach 150–300 µM/L at the expense of red blood cells, since whole blood contains higher levels of glutamate. However, in most cases, the functionality of the BBB is not completely lost. Consequently, the concentration of glutamate that appears after partial destruction of the BBB will be lower than the concentration of plasma glutamate (50 µM/L). The concentration of glutamate in the brain, CSF, and ECF depends on the degree of BBB damage as its determining factor.

As established above, the process of neuronal death provides an explanation for elevated levels of glutamate in the ECF and CSF of the brain shortly after TBI (from the first minutes to the first week). The initial impact from TBI leads directly to cell necrosis from the impact, exacerbated by ischemia, hypoxia, axonal shearing, and gliosis [51,52,53]. Neuronal damage occurs with a massive influx of calcium, which subsequently causes over-excitation and additional release of glutamate [54,55]. This process begins immediately, in the first minutes after injury, and is characterized by significant neuronal death followed by the release of glutamate into the ECF. Cell death lasts up to several hours or days [56,57,58,59], depending on the volume of brain destruction and on the size and duration of cerebral edema and penumbra. This process, however, clearly does not fully account for the occurrence of neuropsychiatric consequences after TBI that manifest themselves months, years, and even decades after the head injury.

### 3.2. The Mechanism of Increase in Brain Glutamate Is Associated with Inflammation [42]

Cell death in TBI patients leads to inflammation in the injured brain (focal brain inflammation), which secondarily causes brain injury through brain edema and neuronal death, even as it binds the damaged limitans and removes cellular debris. However, brain inflammation can also disseminate to other areas of the brain beyond the injured part [42]. Inflammation through glutamate alterations may also be a cause of mood disorders, as patients with mood disorders have been observed to have increased inflammation [38]. Inflammation and alterations in glutamate neurotransmission at the level of the glia increases glutamate and disrupts extrasynaptic signaling. Glutamate diffusion outside the synapse can produce lost synaptic fidelity and specificity of neurotransmission that leads to circuit dysfunction and behavioral pathology [60]. It has also been suggested that inflammation may have an impact on altering behavior of glutamate metabolism, which can cause depressive symptoms such as anhedonia and psychomotor slowing [38].

However, inflammation cannot be the only explanation for psychiatric symptoms following brain injury. If inflammation was closely associated with those symptoms, then conditions with significant neuroinflammation and stress, such as encephalitis, meningitis, and sepsis, would also present with associated neuropsychiatric diseases such as depression, anxiety, aggression, and dementia to a similar degree as after TBI. However, studies show little if any association between those conditions of neuroinflammation and neuropsychiatric disease or show the presence of neuropsychiatric diseases that are secondary to the neuroinflammation itself [61,62].

### 3.3. The Mechanism of Increases in Brain Glutamate Is Associated with Impaired Glutamatergic Recycling and Signaling

Disruption in brain glutamate impairs glutamatergic recycling and signaling [63]. In neurodegenerative diseases, such as Alzheimer’s disease, the system of glutamate recycling is significantly impaired [43,64].

### 3.4. The Mechanism of Increase in Brain Glutamate Is Associated with Prolonged Stress

Glutamate neurotransmission, through its mediation of mood function and stress responses in the brain, has a relationship with the physical biochemical connections of stress and depression [29].

The relationship between increased brain glutamate and stress has also been studied. In one study of induced panic in healthy subjects, a significant rise in brain glutamate was observed in the anterior cingulate cortex [65]. Some studies, however, have shown only a slight correlation between increased glutamate and stress [66] or none at all [67].

### 3.5. The Mechanism of Increase in Brain Glutamate Is Associated with Astrocytic Release of ATP [44]

Astrocytes release ATP, which prompts signaling between neurons and Schwann cells in the peripheral nervous system. When it results from neuronal activity, released ATP can also modulate central synaptic transmission. Glutamate release that occurs during neuronal activity also initiates non-NMDA receptors of astrocytes and ATP release from them, which causes homosynaptic and heterosynaptic suppression. Glutamate, then, may play a role in synaptic modulation [68].

### 3.6. The Mechanism of Increase in Brain Glutamate Is Associated with Other Sources of Elevated Intraparenchymal Glutamate

The intraparenchymal–blood glutamate concentration gradient is governed by normal baseline levels of glutamate in the blood and in the CSF. The difference between these concentrations is close to 50 μM, a level that creates a normal intraparenchymal concentration gradient. The integrity of the BBB preserves this gradient, and alterations in BBB integrity disrupt it. As a result of a variety of brain diseases, including both acute brain injury and chronic disease, increased glutamate levels in the blood and CSF lead to disruptions in the intraparenchymal–blood concentration gradient via compromised BBB integrity [36]. Thus, there are a number of mechanisms that can cause an increase in glutamate in brain tissue. However, with a healthy BBB and well-functioning brain glutamate active transport systems, the brain is able to quickly clear excess glutamate from the brain into the plasma and maintain physiologically low levels of glutamate.

## 4. Disruption of the BBB

A critical process that can induce an increase in brain glutamate in the CSF and ECF is a disruption of the BBB. The destruction of the BBB after brain injury is described as biphasic [69]. The peak of the first phase occurs 5 h after the injury [69], and the second phase peaks in rats after 72 h [70,71,72] and in humans on day 3 [73,74,75] after injury. Recent data show that recovery of the integrity of the BBB in rats can take 1–3 months [76] or even up to 10 months [77], and up to many years after the brain injury in humans [78,79,80]. It is known that damage to the BBB impedes thorough clearance of cerebral glutamate from ECF into the bloodstream, in which excitatory amino acid transporters (EAAT) on endothelial cells underlie the mechanisms for modulating the intraparenchymal–blood glutamate concentration gradient [36].

The integrity of the BBB is a natural limiter of the pathological increase in the levels of ECF/CSF glutamate in the phase of neuronal death and plays a key role in setting the upper level of the concentration of ECF/CSF glutamate in the brain after TBI. However, this is only one aspect of the process. It is the integrity of the BBB that also controls the lower level of the brain glutamate level in all phases of both the healthy and the affected brain after TBI.

In a healthy brain with stable BBB integrity, control of low glutamate levels is maintained by facilitative and active transport systems of the BBB that efficiently remove glutamate from the brain into the blood [81]. When the BBB is destroyed, these systems cannot effectively remove glutamate from the CSF and ECF into the blood and cannot balance its concentration with the blood. Previously, it was believed that the BBB is restored a short time after TBI and after a week its integrity does not significantly differ from those of the control groups without TBI [82]. However, recent data show that disruption of the BBB does not recover within hours, days, or even weeks but continues for many months, years, and even decades after TBI [78,79,80]. Newly developed, highly sensitive methods for determining BBB permeability based on molecular complexes with a molecular size starting from 103 Da [83] register the difference between the control and TBI groups in human and rat populations months and even years after TBI [78,79,80]. These methods are more sensitive and accurate than previous methods to measure BBB permeability, such as with Evans Blue staining, which may explain why a theory of long-term (chronic) glutamate neurotoxicity had not developed until recently [77,78].

Despite a high correlation of sensitivity between nervous tissue and high concentrations of glutamate, studies suggest that a prolonged increase of even 10% of extracellular brain glutamate (considered glutamate neurotoxicity) may induce neurodegenerative processes [12].

**Figure 1:**
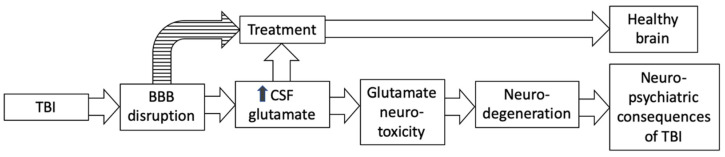
Role of the blood–brain barrier in the mechanism of post-traumatic brain injury neuropsychiatric consequences.

Other processes described above are well documented in the scientific literature and can also increase glutamate levels both locally in brain tissue and in CSF. However, it is the degree of integrity of the BBB that is the key factor controlling the range of glutamate concentration in both healthy and damaged brains. Thus, for the first time, we hypothesize that it is the integrity of the BBB that is the key factor that modulates glutamate in CSF and ECF after brain damage and specifically after TBI, which, via high levels of glutamate, causes neurodegenerative processes which ultimately lead to mental disorders (Figure 1).

## 5. Glutamate Neurotoxicity and Its Association with Neurodegeneration

There is well-established evidence that shows a relationship between glutamate neurotoxicity and neurodegeneration [29]. Glutamate excitotoxicity describes the process of excessive glutamate causing neuronal degeneration and dysfunction, resulting in neurotoxicity [29,84,85]. Its role in mood disorders has informed recent research into new therapeutic modalities, with drugs that target glutamatergic systems proposed as anti-depressants and related therapies [86,87,88].

Increased extracellular glutamate can prompt excitotoxicity, via overaction of ionotropic glutamate receptors, after acute brain insults such as ischemic stroke, cerebral ischemia, TBI, hypoglycemia, and epilepsy [85,89,90,91,92,93,94]. In addition to these acute conditions, a process of chronic glutamate neurotoxicity has also been suggested as a factor in many neurodegenerative diseases, such as amyotrophic lateral sclerosis, Alzheimer’s disease, and Huntington’s disease [92]. In these cases, it is possible that chronic excitotoxicity occurs in diseases where nerve cell death occurs over a longer period of time, in which neurons exposed to glutamate at higher-than-normal levels can gradually lead to cell death or impaired neuroplasticity [92]. We hypothesize that therapeutic modalities for these diseases may work by specifically restoring glutamatergic homeostasis through stimulating glutamate uptake and releasing extracellular glutamate. The process of glutamate neurotoxicity leading to neurodegeneration leads to new possibilities for treatments for acute and chronic neurological diseases that target the glutamatergic system.

## 6. New Treatment Strategies for Neuropsychiatric Consequences of TBI Associated with the Hypothesis of Impaired BBB Permeability and Brain–Blood Glutamate Equilibrium

Immediately following TBI, the concentration of cerebral glutamate increases, then decreases. However, it does not reduce to normal levels; excess levels can persist for months or years [74,75]. As noted above, an excess of glutamate in CSF and ECF over time causes neurodegenerative processes that ultimately lead to the development of neuropsychiatric pathology (Figure 1). Modulating excess glutamate, therefore, is a critical factor in limiting the spread of brain damage. According to our hypothesis, BBB permeability is a key factor controlling the upper and lower levels of CSF and ECF glutamate. Therefore, we propose two promising strategies to reduce the level of brain glutamate. The first strategy involves the treatment and reconstruction of the BBB. The goal of the second strategy is to reduce CSF and ECF glutamate levels by lowering plasma glutamate levels.

### 6.1. Treatment Aimed at Recovery of the Integrity of the BBB [95,96]

Treatments to restore BBB function and integrity consist of several approaches (Table 1) [95]. However, some treatments solely aimed at restoring the BBB have not been shown to be effective in treating neurodegenerative diseases [97,98]. Thus, this approach alone may be inadequate in treating symptoms of BBB dysfunction.

### 6.2. Reducing Post-Injury Glutamate Excess Based on Manipulation of Brain–Blood Glutamate Equilibrium

Excess brain glutamate can also be modulated by changing the brain–blood glutamate equilibrium and sending excess glutamate from the brain’s interstitial fluid (ISF) into the body’s circulatory system [99]. Glutamate transporters on the endothelial cells of brain capillaries enable extrusion of glutamate from ISF [37,100]. Synaptic glutamate receptors are not directly stimulated or impeded through this process, allowing mechanisms of learning to continue.

Treatments shown in a rat model to lessen the early neuroanatomical and neurological detriments caused by TBI include administration of the enzymes glutamic oxaloacetic transaminase (GOT), serum glutamic pyruvic transaminase (GPT), and their co-substrates oxaloacetic acid (OxAc) and pyruvate, which reduces excess brain glutamate by altering the balance between blood and brain glutamate [101,102,103]. This reduction in glutamate is referred to as blood glutamate scavenging (BGS).

It has been shown that low levels of GOT and GPT are associated with poor neurological outcomes following stroke, whereas high GOT and GPT levels correlate with a better neurological outcome [104,105]. The enzymes GOT and GPT use glutamate as a substrate and pyridoxal phosphate as a cofactor to reversibly convert glutamate into the inactive metabolite 2-ketoglutarate.

This process prompts blood glutamate levels to fall below basal levels, causing a much steeper gradient of glutamate levels between the ISF or CSF and blood. To attain an equilibrium again, glutamate is moved from the brain to the blood, thus reducing the high level of glutamate in the brain. As long as glutamate stays at a low level in the blood, this brain-to-blood efflux will continue. To maintain the proper functionality of GOT and GPT to convert glutamate into 2-ketoglutarate, their respective substrates OxAc and pyruvate must be administered at doses at least double their Km values. The conversion of glutamate to 2-ketoglutarate is reversible. Thus, upon glutamate transformation via an enzymatic reaction into 2-ketoglutarate, there is an accrual of 2-ketoglutarate which can cause the enzyme to convert 2-ketoglutarate into glutamate. Thus, it is advantageous to break down 2-ketoglutarate fully to ensure the continual metabolism of glutamate. 2-ketoglutarate (with lipoamide) is reversibly metabolized by the enzyme 2-ketoglutarate dehydrogenase to S-succinyldihydrolipoamide and carbon dioxide [74]. The increased concentration gradient between blood and brain glutamate accelerates the efflux of glutamate from brain to plasma, thereby minimizing excitotoxicity caused by excess brain glutamate.

OxAc and pyruvate infusions reduce the severity of neurological deficits and symptoms of depression in rats after stroke [106] and traumatic injury in the brain [46]. The therapeutic effect of pyruvate on aggression and anxiety symptoms in a rat model of TBI has been observed in our laboratory (data yet to be published). The efflux of glutamate with pyruvate or OxAc is blocked by adding exogenous glutamate, demonstrating the dependence of the scavenging method on lowering blood glutamate [37,103]. GOT and GPT likely enhance the efflux of glutamate from the brain and blocking the function of GOT and GPT exacerbated the behavioral and morphological deficits following ischemia [107].

OxAC, pyruvate, GPT, and GOT have been proposed as treatments for TBI and stroke [46,106,108,109,110]. The safety of OxAc and pyruvate has been shown in elderly patients [111,112,113,114,115,116,117,118,119,120], and its potential in alleviating symptoms of anxiety, depression, suicidal ideation, and aggression in women with PMS was suggested in a clinical trial [121].

**Table 1:** Treatments to restore blood–brain barrier function and integrity.

Intervention	Description
Targeting paracellular permeability	Targeting junction molecules (adherens junctions, tight junctions), or their regulators (microRNA, transcription factor) in order to limit or reverse paracellular permeability [95,122]. Examples include chelerythrine chloride [123].
Targeting transcellular permeability	Inhibition of transcytosis in brain endothelial cells, important to maintain neurological function and BBB integrity [95,124,125].
Restoring efflux transporter activity	Restoring efflux transporter activity, such as ATP-binding cassette (ABC) transporters [95,126], important for clearing neurotoxins from the brain.
Repair of the neurovascular unit	Reestablishing normal function of the neurovascular unit (neurons, astrocytes, endothelial cells, pericytes, and the basal lamina), by restoring microvascular bed cerebral blood flow, limiting neuronal death, and promoting neurogenesis and angiogenesis [127]. Examples include bone-marrow-derived mesenchymal stem cells (MSCs) [128], pericytes [129,130], endothelial progenitor cells (EPCs) [131], neural and vascular progenitor cells [132,133], bone-marrow-derived macrophages [134], and vascular endothelial growth factor (VEGF) [135].
Targeting inflammation	Targeting inflammation and downstream sequalae to restore the BBB. Examples include COX-2 inhibition [136], AQP4 inhibition [123], docosahexaenoic acid (DHA) [137], inhibition of Na-K-Cl cotransporter [138], and bone marrow mononuclear cells (MNCs) [139,140].
Matrix metalloproteinases (MMP)	Limiting pathologically elevated MMP expression elevated after brain insult [127]. Examples include progesterone [141], TGF- β1 [142], exendin-4 [143], melatonin [144], regulatory T cells [145], EP1 antagonists [146], and minocycline [147].

## 7. Other Factors That Play a Role in the Pathophysiology of TBI

Although we propose in this paper that a disruption to BBB integrity is associated with neurological deterioration, other factors may also play a role. These factors include age at the time of injury, hormonal status, medical history, and environmental factors with an impact on general stress [148]. While a discussion of these other factors is beyond the scope of this manuscript, we anticipate future research will provide more information on the possibility of multifactorial pathophysiology of neuropsychological symptoms of brain injury.

## 8. Conclusions

There are several mechanisms that potentially influence an increase in brain glutamate associated with TBI: (i) neuronal death; (ii) destruction of the BBB; (iii) inflammation; (iv) impaired glutamatergic recycling and signaling; (v) prolonged stress; (vi) astrocytic release of ATP; and (vii) other sources of elevated intraparenchymal glutamate. These mechanisms start in the first minutes after brain injury and continue for many years and even decades after TBI.

Regardless of how much glutamate is released into the CSF and ECF compartment via the above mechanisms, BBB disruption is one component that determines the maximum levels of CSF and brain ECF glutamate. The degree of disruption of BBB integrity controls the glutamate range in both healthy and TBI brains based on two parameters: BBB permeability and the ability of facilitative and active transport systems to remove glutamate into the bloodstream.

Thus, we novelly hypothesize that it is the integrity of the BBB that is the key factor in the context of the control of glutamate in CSF and ECF after brain damage. Specifically, this occurs after TBI, in which neurotoxicity involving high levels of glutamate causes neurodegenerative processes which ultimately lead to mental disorders. Consequently, targeting reparation of BBB integrity through controlling increased glutamate may together have a significant impact on neuropsychological symptoms of acute and chronic brain conditions. We anticipate that future research will explore the role of the BBB as a central location for the development of neuropsychiatric consequences of TBI.

## Data Availability

Not applicable.
